# A Rare Presentation of Lymph Node Metastasis of VIPoma After Three Years of Resection: A Case Report

**DOI:** 10.7759/cureus.57628

**Published:** 2024-04-04

**Authors:** Oğuzhan Şal, Katsunori Sakamoto, Kei Tamura, Masahiko Honjo, Yusuke Nishi, Takahiro Hikida, Akimasa Sakamoto, Naotake Funamizu, Kohei Ogawa, Yasutsugu Takada

**Affiliations:** 1 Department of General Surgery, Istanbul University Faculty of Medicine, Istanbul, TUR; 2 Department of Hepato-Biliary-Pancreatic Surgery, Ehime University Graduate School of Medicine, Ehime, JPN; 3 Department of Hepato-Biliary-Pancreatic and Breast Surgery, Ehime University Graduate School of Medicine, Ehime, JPN

**Keywords:** pancreatic neuroendocrine tumors, lymphadenectomy, lymph node metastasis, metachronous, vipoma

## Abstract

Vasoactive intestinal peptide-producing tumor of the pancreas (VIPoma) is one of the rarer subtypes of neuroendocrine tumor (NET) of the pancreas. It usually represents intractable diarrhea, weight loss, and electrolyte abnormalities secondary to diarrhea. The most common site of metastasis of VIPoma is the liver. Furthermore, lymph node metastasis (LNM) is rare, and no metachronous LNM with a resectable situation has been reported before. A 60-year-old male patient (height: 181 cm, body weight: 74 kg) with a history of operated pancreatic VIPoma three years ago was referred to our department due to the detection of lymphadenomegaly which was suggestive of lymph node metastasis by routine follow-up computed tomography (CT). Preoperative CT showed a lymph node on the left side of the abdominal aorta and caudal side of the left renal vein with a size of 1 cm. Lymphadenectomy was performed without significant complications and blood loss. This is the first report of metachronous LNM in a patient with operated VIPoma. Although much rarer than solid organ metastasis of VIPoma, LNM in these patients can also be seen synchronously and metachronously. Close follow-up and vigilance are key to preventing recurrence-related morbidity and mortality in these patients.

## Introduction

Neuroendocrine tumors (NETs) of the pancreas are rare malignant neoplasms of the pancreas second to adenocarcinoma. Furthermore, vasoactive intestinal peptide-producing tumor of the pancreas (VIPoma) represents a significantly smaller portion of NET of the pancreas when compared to insulinoma and non-functional NET of the pancreas [1]. Despite being rare, it presents with insidious and comorbid symptoms such as intractable diarrhea and electrolyte abnormalities such as hypokalemia and hyponatremia due to the oversecretion of vasoactive intestinal peptide (VIP) from the tumor [2]. Due to its insidious onset, the disease is usually detected in late stages with distant organ metastasis with lymph node metastasis being extremely rare [3]. In this paper, we represent the first case of VIPoma with metachronous lymph node metastasis in a 60-year-old patient three years after the resection of the primary tumor.

## Case presentation

**Figure 1:**
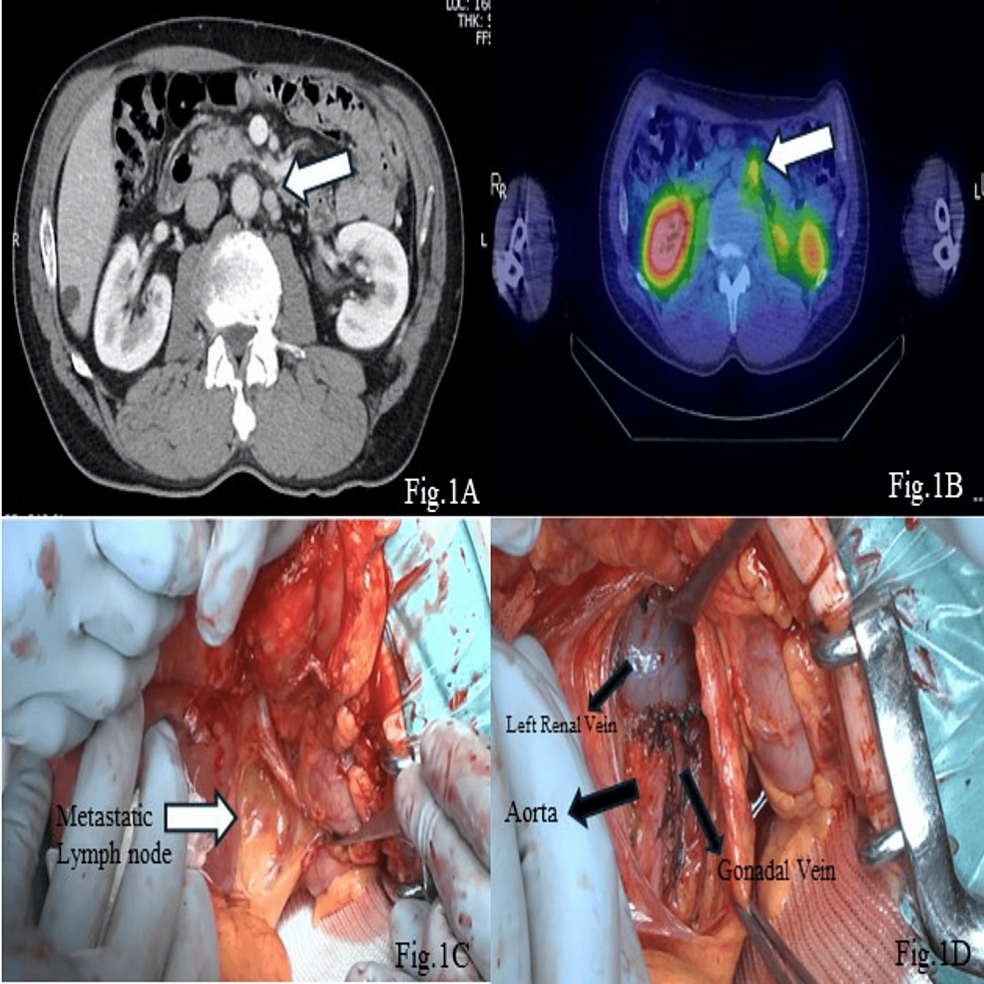
Preoperative images of the metastatic lymph node and anatomical location of the metastatic lymph node. Figure 1A: CT image of the mass (shown with white arrow). Figure 1B: Somatostatin receptor scintigraphy image of the mass (shown with white arrow). Figure 1C: Metastatic lymph node in the para-aortic station (shown with white arrow). Figure 1D: Gonadal vein, left renal vein, and aorta visible after resection of the metastatic lymph node.

**Figure 2:**
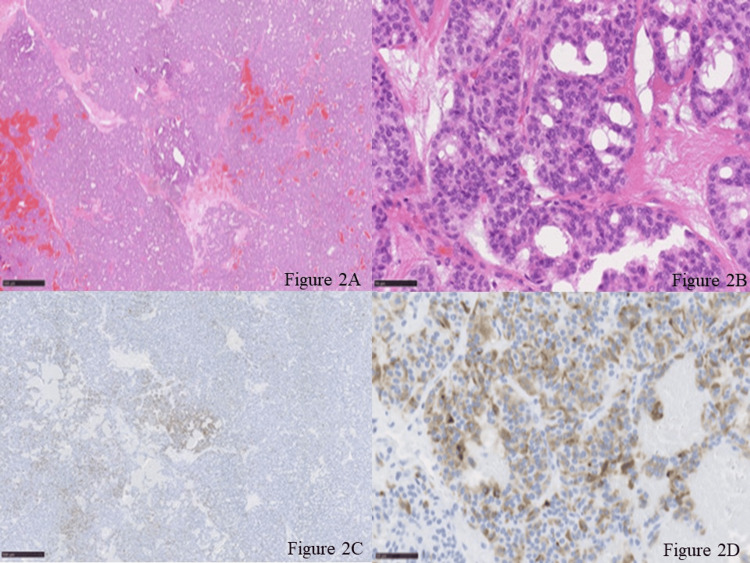
Pathological examination of the metastatic lymph node with hematoxylin and eosin stain and VIP immunohistochemistry. Figure 2A-2B: Hematoxylin-eosin staining of the lymph node (×40 and ×400). Tumor cells with round nuclear display an organoid growth pattern surrounded by capillaries. Immunohistochemical staining of chromogranin A, synaptophysin, and CD56 are positive, and the tumor shows a Ki-67 of 2%. Figure 2C-2D: VIP immunohistochemical staining (×40 and ×400). Positive staining of VIP is shown. VIP: vasoactive intestinal peptide

A 60-year-old male patient with a history of operated pancreatic tail VIPoma three years ago with a WHO classification (2017) of NET (G2) without neoadjuvant chemotherapy and radiotherapy was referred to our center due to lymphadenomegaly in para-aortic stations which suggested lymph node metastasis on routine follow-up CT scan. The patient was diagnosed with watery diarrhea-hypokalemia-achlorhydria (WDHA) syndrome due to VIPoma of the tail of the pancreas at 57 years old. At the time of the diagnosis, the value of serum potassium was 1.6 mEq/L by frequent water diarrhea, and he has massive fatigue. He also had rhabdomyolysis and poor glucose intolerance. He underwent an open distal pancreatectomy with regional lymphadenectomy for VIPoma at another hospital, and he became free from symptoms. The diameter of the tumor was 9 cm, and the surgical margin was negative. The tumor was diagnosed as NET (G2) by WHO classification with a Ki-67 of 4%. The pathological findings of the primary lesion showed positive VIP in immunohistochemistry (IHC) staining. No lymph node metastasis was seen at that point in time: by preoperative somatostatin receptor scintigraphy and early postoperative follow-up scans. Then he was followed up by our hospital thereafter from 58 years old with the management of diabetes mellitus. When he was 60 years old, CT showed a 1-cm-diameter lesion in the para-aortic region, on the left side of the abdominal aorta and caudal side of the left renal vein (Figure 1A). Although he was free from symptoms that he had experienced in his first surgery, ^111^ In-pentetreotide somatostatin receptor scintigraphy detected accumulation in the para-aortic lymph nodes; there was no uptake in other lymph node stations or distant organs. These findings were in concordance with CT (Figure 1B). In addition to those findings, local recurrence of the tumor within the lymph node stations became an indication for resection for the improvement of overall survival. The VIP value was not examined at both the time of diagnosis of the primary lesion and the metachronous lymph node metastasis, and no interventional radiological modality was used during the diagnosis and management. The patient was placed in a supine position, and an open approach was selected. Pathologic lymph node was observed in the para-aortic station, and lymphadenectomy of the positive lymph nodes detected preoperatively was performed without extended lymphadenectomy (Figure 1C-1D). The operation lasted 106 minutes, and the estimated intraoperative blood loss was 30 ml. No perioperative transfusion was performed, and the patient did not need intensive care unit care. There was no significant complication in the postoperative period. The patient was discharged in POD 6 without any complications. Pathological examination of the specimen revealed that, of five lymph nodes, two lymph nodes had VIPoma metastasis (10 mm and 3 mm). IHC staining of the lymphadenectomy specimen revealed a NET (G2) with a Ki-67 of 2% (Figure 2A-2D). The patient is free from recurrence at three months after the operation.

## Discussion

VIPoma is one of the rarest forms of pancreatic NETs with an incidence of less than 0.22 cases per 100,000 [4]. It is usually diagnosed at late stages due to its insidious onset and the non-specific nature of the symptoms [5]. The disease usually presents itself with intractable watery diarrhea, electrolyte abnormalities such as hyponatremia and hypokalemia, and metabolic acidosis which initially lead to investigation of other etiologies of watery diarrhea such as colitis and small bowel disorders [3]. Due to late diagnosis of the disease, it becomes an already distant organ metastasis, mostly liver, and in late stages, this limits resectability and decreases survival [5]. On the other hand, the mainstay of therapy in resectable VIPoma cases is resection of the primary tumor and if possible metastasectomy. Contrary to the distant organ metastasis of VIPoma, lymph node metastasis (LNM) of the disease is quite rare; in the literature, Ueda et al. reported the only case of LNM of VIPoma which represented synchronous metastasis of the disease to para-aortic lymph node stations. In the report, a 72-year-old female patient presented with classical symptoms of VIPoma, and preoperative CT showed a 55 mm tumor located in the pancreatic tail with para-aortic lymphadenomegaly. Furthermore, fine needle aspiration biopsy revealed a VIPoma; distal pancreatectomy and para-aortic lymphadenectomy were performed. Short-term follow-up of the patient revealed no recurrences or distant organ metastasis [6]. On the other hand, in our case, we represent a metachronous LNM of VIPoma, which was detected three years after the resection of the primary tumor and surgically removed in order to prolong survival. In addition, the definitive diagnosis of the primary tumor was known and allowed conscious presumptions of recurrence. Furthermore, it has been suggested by the National Comprehensive Cancer Network that in neuroendocrine tumors, for patients with liver, lung, and intra-abdominal lymph node metastasis, surgical resection is indicated if the lesions are curatively resected for both synchronous and metachronous metastasis [7]. In addition, there are studies showing that an aggressive surgical approach in patients with recurrences of neuroendocrine pancreatic tumors, although not significantly, improves overall survival [8]. Unlike the classical approach to lymphadenectomy in oncological settings, we only performed a restricted lymphadenectomy due to the only metastatic region affected being para-aortic stations and close follow-up availability. Although the option to perform extended lymphadenectomy of para-aortic lymph nodes was considered for the present case, there has been no evidence of positive prognostic impact regarding extended para-aortic lymph nodes in gastric cancer [9]. Extended para-aortic lymphadenectomy does not only have a positive prognostic impact, but it also causes various severe complications [9]. Since there has been no evidence of extended para-aortic lymphadenectomy in a neuroendocrine tumor, we only removed the positive lymph stations which consisted of five lymph nodes, of which two of them were positive. During the follow-up period, the patient had no symptoms such as watery diarrhea, weight loss, or unspecified abdominal pain to suggest recurrence; therefore, close follow-up and radiological studies were the main factors allowing early detection and lymphadenectomy. 

## Conclusions

Although VIPoma is one of the rarer forms of NET of the pancreas, it presents with debilitating symptoms such as intractable diarrhea and electrolyte abnormalities. Lymph node metastasis of VIPoma is extremely rare; however, close follow-up and vigilance are vital for detecting early recurrences and distant organ metastasis.
